# Osteogenic differentiation of mesenchymal stem cells cultured on PLLA scaffold coated with Wharton's Jelly

**DOI:** 10.17179/excli2016-741

**Published:** 2017-05-23

**Authors:** Marziehsadat Ahmadi, Ehsan Seyedjafari, Seyed Jalal Zargar, Gebremariam Birhanu, Ali Zandi-Karimi, Bahareh Beiki, Kadriye Tuzlakoglu

**Affiliations:** 1 Department of Cell and Molecular Biology, School of Biology, College of Science, University of Tehran, Iran; 2 Department of Biotechnology, College of Science, University of Tehran, Iran; 3 Department of Pharmaceutics, Faculty of Pharmacy, Tehran University of Medical Sciences, International Campus (TUMS-IC), Tehran, Iran; 4 School of Pharmacy, College of Health Sciences, Addis Ababa University, Ethiopia; 5 Department of Polymer Engineering, Yalova University, Turkey

**Keywords:** Wharton's Jelly, poly-L-lactic acid, coating, electrospinning

## Abstract

Poly-L-lactic acid (PLLA) electrospun nanofiber scaffold is one of the most commonly used synthetic polymer scaffolds for bone tissue engineering application. However, PLLA is hydrophobic in nature, hence does not maintain proper cell adhesion and tissue formation, moreover, it cannot provide the osteo-inductive environment due to inappropriate surface characteristic and the lack of surface motives participating in the first cellular events. To modify these shortcomings different approaches have been used, among those the most commonly used one is coating of the surface of the electrospun nanofiber with natural materials. In this work Wharton's jelly (WJ), a tissue which surrounds the umbilical cord vessels, reaches in high amounts of extracellular matrix (ECM) components mainly; collagen, hyaluronic acid and several sulphated glycosaminoglycans (GAGs) were used to cover the surface of electrospun PLLA nanofiber scaffolds. The surface morphology of the nanofiber scaffold was evaluated via scanning electron microscope, and the *in vitro* osteogenic differentiation potential was determined by MTT assay and common osteogenic marker tests such as alkaline phosphatase (ALP) activity and calcium deposition tests. Coating of WJ could not change the surface morphology and diameter of the nanofibers. However, WJ-PLLA scaffolds showed higher proliferation of human mesenchymal stem cells (MSC) than tissue culture plate (TCP) and pristine PLLA scaffolds, moreover, WJ-PPLA scaffold demonstrated significant alkaline phosphatase activity and calcium mineralization than either TCP or PLLA nanofiber scaffolds.

## Introduction

Bone is self-regenerative tissue, however, in case of large bone defects after injury or tumor resection self-regeneration is impossible. In this case, it is possible to repair large bone defects with the help of tissue engineering (Buser et al., 1993[8]). The base for bone regeneration and repair using tissue engineering is the formation of bone *ex vivo* using cells and other factors that will stimulate bone growth on tissue engineered scaffolds (Seyedjafari et al., 2011[30], Shabani et al., 2014[31]; Ramezanifard et al., 2016[26]). Tissue engineered scaffold is one of the main component and backbone of tissue engineering, whose primary function is to support cell proliferation and differentiation (Hench and Polak, 2002[17]). The ideal tissue engineering scaffold should mimic the structure of natural extracellular matrix (ECM), which is composed of collagen nanofibers (50-500 nm). Nanofibers could also facilitate the osteogenic differentiation of osteoblasts and mesenchymal stem cells (MSCs), which was suggested via a RhoA-Rock signaling pathway relating to cell adhesion, spreading, and osteogenic differentiation (Duan et al., 2015[12]).

There are different biopolymers and biomaterials that can be used to make a scaffold in tissue engineering (Amjadian et al., 2016[3]). Scaffolds made from synthetic biomaterials such as metals, polymers, porous ceramics, hydroxyapatite, collagen sponge or hydrogels, and composites can be used as a bone substitution for restoration of large bony defects (Shin et al., 2005[32]; Agarwal et al., 2008[1]; Glowacki and Mizuno, 2008[13]; Schofer et al., 2011[28]). In these scaffolds, nanoscaffolds have innumerable benefits (Zhang et al., 2005[36]). They are believed to mimic the ECM (Woo et al., 2007[35]) and help the differentiation of hMSC towards osteoblasts. Nevertheless, their effect depends on the physico-chemical composition of the nanofibers (Schofer et al., 2011[29]).

Synthetic aliphatic polyesters such as poly (glycolic acid) (PGA), poly (lactic acid) (PLA), their copolymers (e.g., PLGA), and polycaprolactone (PCL) are the most commonly used polymers for tissue engineered scaffolds (Agrawal and Ray, 2001[2]).

Among those PLLA is one of the poly alpha hydroxyl esters which can be used for different applications including; bone graft and drug delivery because of its biocompatibility, biodegradability, structural supporting of damaged tissue through the gradual transition pressure to defect site as a result of gradual degradability and the capability of being modified physically, chemically and biologically (Lin et al., 2006[20]). However, PLLA and other polymers are mostly hydrophobic in nature, hence do not maintain proper cell adhesion and tissue formation (Mattii et al., 2008[23]). Moreover, PLLA cannot provide the osteoinductive environment due to inappropriate surface characteristic and the lack of surface motives participating in the first cellular events (Amjadian et al., 2016[3]) as evidenced by an initial down regulation of genes associated with the osteoblast lineage on PLLA nanofibers (Schofer et al., 2011[29]).

A variety of approaches were used to overcome these drawbacks without altering the bulk properties such as; plasma treatment, graft techniques, and the use of natural polymers in the form of composite scaffold or coating. Among them, use of natural polymers becomes an effective approach (Amjadian et al., 2016[3]). Naturally occurring polymers extracted from the native extracellular matrix (ECM) have been used to modify the synthetic materials. Considering that major ECM components are collagens (Schofer et al., 2011[29]; Mattii et al., 2008[23]), elastin, and glycosaminoglycans (GAGs), one or more of these components could be profitably used in the preparation of scaffolds depending on the tissue to be regenerated (Daamen et al., 2008[11]) because they are naturally within the ECM and mimic it more likely and mediate the cell anchoring and signaling involved in cell spreading, migration, mitosis, differentiation and death (Ma et al., 2005[21]).

Wharton's jelly (WJ) is a tissue, which surrounds the umbilical cord vessels. It has a very low number of cells but high amounts of ECM components mainly; collagen, hyaluronic acid and several sulphated glycosaminoglycans (GAGs) (Sobolewski et al., 2005[34]; Malkowski et al., 2007[22]). It is a collagen-rich tissue (Gogiel et al., 2003[14]); in other words, collagen is a main component of WJ ECM, it constitutes above 50 % of the weight of defatted, dry tissue. WJ is also rich in GAGs, mainly hyaluronic acid that constitutes almost 70 % of total GAG content (Sobolewski et al., 1997[33]). The large amount of hyaluronate makes this tissue highly hydrated (Romanowicz and Bankowski, 2010[27]). Moreover, WJ is rich in a variety of fibrous and interstitial proteins, and signaling molecules, including glycosaminoglycans (GAGs), proteoglycans, (Chan et al., 2009[9]) and peptide growth factors (Romanowicz and Bankowski, 2010[27]), all of which could facilitate cellular attachment, proliferation, infiltration, and differentiation, leading to constructive ECM remodeling and tissue reconstruction (Chan et al., 2009[9]). In addition, WJ provides a natural microenvironment that preserves MSC properties (Hao et al., 2013[16]). Therefore, we here hypothesized that coating of WJ on PLLA electrospun nanofiber scaffolds would be valuable for MSC culture expansion *in vitro *and would preserve MSCs properties by improving the drawbacks associated with PLLA electrospun nanofiber scaffolds for ideal bone tissue engineering. This study demonstrated that coating of WJ can greatly improve the osteogenic potential of PLLA nanofiber scaffolds.

Based on these facts, WJ extract from the human umbilical cord was used to coat PLLA electrospun nanofiber scaffold (WJ-PLLA) for bone tissue engineering application. WJ-PLLA electrospun nanofiber scaffold was fabricated with the aim of evaluating the *in vitro* osteogenic differentiation potential of MSCs cultured on it. As to our knowledge no work has been done before that contains PLLA electrospun nanofiber scaffolds coated with WJ for bone tissue engineering application. 

## Materials and Methods

Chemicals and reagents used in the study were analytical reagent grade.

### Fabrication of PLLA nanofiber scaffold

To prepare the PLLA nanofiber scaffold, 7.3 % (w/v) solution of PLLA (Mw = 157000, Sigma-Aldrich) in chloroform and dimethylformamide (DMF) (Merck, Germany) was put into a syringe which has a needle, and was connected to a voltage of 18 kV between the needle and collector. The distance between the needle tip and the collector was15 cm with a mass flow rate of 0.5 ml/h and sprayed in the form of nanofibers on the cylinder collector covered by aluminum foil and finally the nanofibrous membranes were detached and placed in vacuum drying at room temperature.

### Preparation of human umbilical Wharton's jelly extract

It was done according to previous protocols (Hao et al., 2013[16]), briefly: Human umbilical cord (UC) samples were obtained after the delivery of normal-term babies and were collected in a transfer medium of phosphate-buffered saline (PBS) and 50 IU heparin, then maintained at 4 °C until processing within 24 h of collection. UCs were washed three times with PBS, and the umbilical veins were rinsed with PBS to remove contaminating red blood cells. UCs were cut into 1 cm segments, and UC arteries, veins and amnion were removed. The gelatinous tissue was excised and minced into 0.5-1 mm^3^ pieces. Equal volumes of PBS were used to swell the tissue pieces with constant shaking at 4 °C for 48 h, and the tissue pieces were subjected to 5 freeze-thaw cycles (-80 °C to 37 °C). Samples were ruptured by high-speed dispersion on ice for 10 min and homogenized with a glass homogenizer on ice. Tissue homogenates were centrifuged at 10000 x g for 30 min to remove debris. Finally, the supernatants that were WJ extract was collected.

### Coating of Wharton's jelly

Initially to make a solution of WJ, 1 mg of WJ was dissolved in 9 ml of deionized water and well dispersed using an ultrasonic bath for 20 min, then plasma treated scaffolds were immersed in the WJ aqueous solution overnight, well rinsed with deionized water and dried in vacuum.

### Scanning electron microscopy

The surface morphologically of the nanofibers was identified via a scanning electron microscope (SEM). Small pieces of the scaffolds were placed on a surface and coated with gold using a sputter coater, then the specimens were affected by electron irradiation and finally the images were detected.

### Isolation and characterization of adipose derived mesenchymal stem cells

Isolation of adipose-derived MSC was achieved by treating the adipose tissue with collagenase and trypsin (GIBCO) under shaking at 37 °C and centrifuging after rinsing it with PBS including penicillin and streptomycin (GIBCO) 2 or 3 times. Finally, the isolated cell pellet was resuspended in basal medium (DMEM with 10 % FBS (GIBCO)) and transferred to the flasks in an incubator until reaching the 80 % confluence.

### Cell seeding

For cell seeding, the scaffolds were punched to 1.5 cm diameter circular membranes and sterilized with UV for 20 minutes, then placed in 24 well tissue culture plate and consequently these scaffolds were washed with PBS twice and finally incubated with a basal medium including antibiotics for 24 hours. An initial density of 5000 cells per well for cell viability and proliferation and 10,000 cells per well for differentiation investigations were cultured on the scaffolds. The cell-loaded scaffolds were refreshed every 2 days with basal medium for investigation of the cell attachment and proliferation. For differentiation assessments, this procedure was repeated after reaching 80 % confluence with complete osteogenic medium (basal medium supplemented with dexamethasone, ascorbic acid and beta-glycerophosphate). 

### MTT test

The cell affinity which is defined with adhesion and proliferation of cultured stem cells was investigated through MTT assay. In this method the incubated cell-loaded scaffolds were refreshed with DMEM including 10 % MTT (Sigma-Aldrich) solution after 24 hours for cell adhesion assessment and on days 1, 4, and 7 for cell proliferation assay and incubated again for 3-4 hours in order to oxidize this substrate in the living cells by mitochondrial dehydrogenases.

Then the supernatant was replaced with DMSO (Merck, Germany) as the solvent of the dark blue formazan crystals formed in the live cells and the absorbance of purple solution was read via spectrophotometer (Bioteck) at 570 nm. Finally, the cell numbers were calculated using a standard curve.

### ALP activity

To measure alkaline phosphatase (ALP) activity, first in order to extract the total protein of cells and prevent the extracted proteins from degradation 250 ml RIPA buffer and protease inhibitor were used. The lysate was shaken at 4 °C and centrifuged at 15,000 rpm for 15 min to sediment cell debris. Finally, the supernatant was collected and the ALP activity was measured by using an ALP assay kit (Parsazmoon, Iran) having ρ-nitrophenyl phosphate as the substrate. The activity of the enzyme (IU/L) was normalized against the total protein (mg/dl).

### Calcium content assay

In order to extract the calcium depositions, the cell-loaded scaffolds were homogenized in 0.6 N HCl (Merck, Germany) and then were shaken at 25 °C for 1 hour, eventually, the calcium content was calculated through a calcium content assay kit (Parsazmoon, Iran) and cresolphtalein complex one interaction. Furthermore, the standard curve of optical density (OD) versus concentration was estimated by using a serial dilution of standard solution in the kit.

### Alizarin Red S Staining

To observe the mineralization of the extracellular matrix the cells were stained with Alizarin red S as follows; first, the samples were fixed in ice-cold 70 % ethanol for 1 hour at 4 °C and then stained with 1.36 % Alizarin red S for 1 hour at room temperature and finally washed with PBS. The images under a light microscope were taken.

### Statistical analysis

The results given are representative of three independent experiments. Data were presented as means ± standard deviation. Statistical comparisons were done using one-way analysis of variance (ANOVA). P values <0.05 were considered as significant.

## Results

### Scaffold surface morphology characterization

**Figure 1:**
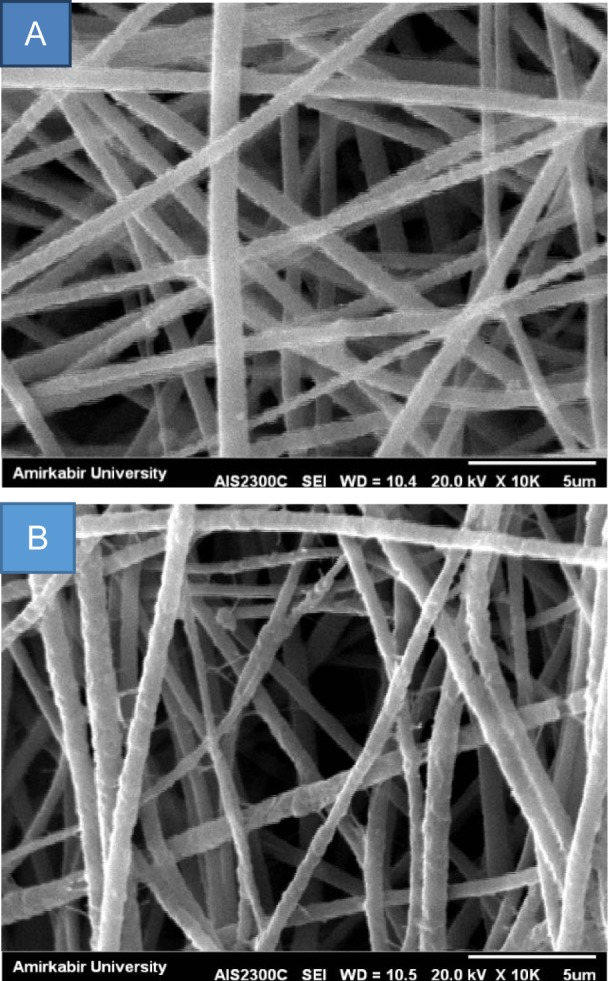
Scanning electron microscope images of electrospun nanofiber scaffolds (A), PLLA (B) WJ- PLLA

As illustrated in Figure 1(Fig. 1), both WJ-PLLA and uncoated scaffolds showed a porous structure with interconnected pores and uniform smooth random-oriented nanofibers. Coating of WJ did not block the pores at the surface of the PLLA scaffolds.

### Cell attachment and proliferation

**Figure 2:**
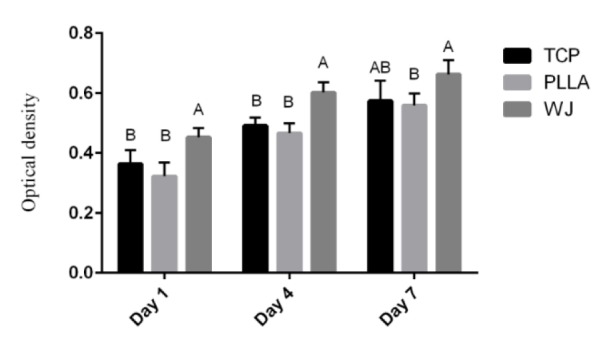
Proliferation of MSC on scaffolds (PLLA and WJ coated PLLA) and TCP. The results given are representative of three independent experiments.

To evaluate the potential of PLLA and WJ-PLLA as tissue-engineered scaffolds, as well as to investigate their effects on stem cell proliferation, cells were cultured on surfaces of nanofibers and their proliferation, infiltration, and osteogenic differentiation were investigated *in vitro*. The MTT result as shown in Figure 2(Fig. 2) demonstrated an increasing pattern of cell population during the period of study, for all 3 scaffolds, i.e. PLLA, TCP and WJ-PLLA nanofiber scaffolds. Starting from day 1 till day 7, the proliferation of MSC in WJ-PLLA scaffold was much better than uncoated PLLA scaffolds and TCP (control). In all the days, stem cells cultured in WJ-PLLA scaffolds showed a significant increase in proliferation than TCP and uncoated scaffolds (P value < 0.05). On day 1 and day 4 cells cultured on WJ-PLLA scaffolds showed a significant proliferation rate (P value < 0.05) than either TCP or uncoated PLLA scaffolds but there was no significant difference in proliferation between cells cultured in TCP and PLLA scaffolds. On day 7, the rate of a proliferation between WJ-PLLA and uncoated PLLA scaffolds was significantly different (P < 0.05), WJ-PLLA scaffold showed great proliferation, but there was no significant difference between WJ-PLLA nanofiber scaffolds and TCP, and between TCP and uncoated scaffolds.

### Osteogenic differentiation of stem cells

**Figure 3:**
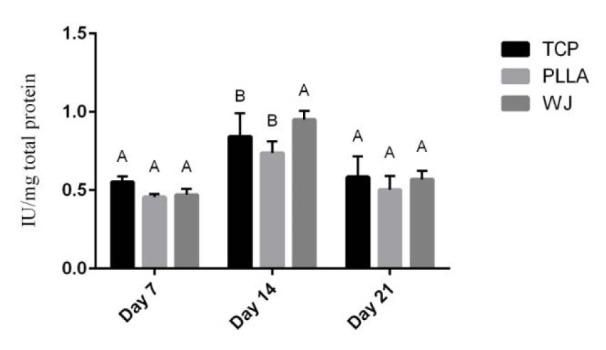
Alkaline phosphatase (ALP) activity of stem cells of PLLA scaffolds, WJ coated PLLA scaffolds and tissue culture plate (TCP) on 7^th^, 14^th^ and 21^st^ day, during osteogenic differentiation. The results given are representative of three independent experiments.

**Figure 4:**
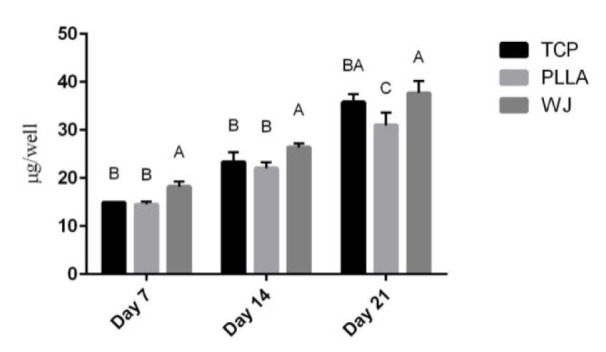
Total calcium content of stem cells on scaffolds (PLLA and WJ coated PLLA) and tissue culture plate (TCP) on 7^th^, 14^th^ and 21^st^ day of osteogenic differentiation. The results given are representative of three independent experiments.

The most common methods to evaluate osteogenic differentiation of stem cells on scaffolds are determination of ALP activity and total calcium content. As shown in Figure 3(Fig. 3), the ALP activity increased until day 14, then decreased on day 21 (P <0.05). On day 7 and 21, there was no significant difference in ALP activity of the cells on the two scaffolds and TCP, and between each scaffold. However, on the 14^th^ day, WJ-PLLA nanofiber scaffolds showed a significant (P<0.05) activity of stem cells during osteogenic induction than pristine PLLA scaffolds and TCP but there was no significant difference between uncoated scaffolds and TCP. Moreover, the evaluation of calcium content (Figure 4(Fig. 4)) showed an increase in calcium content or mineralization in both PLLA and WJ-PLLA scaffolds and TCP, while WJ-PLLA scaffolds gave higher calcium content in the three consecutive weeks than either TCP or PLLA scaffolds. The highest mineralization was observed from WJ-PLLA scaffolds on day 21. On the 7^th^ and 14^th^ day, the mineralization of WJ-PLLA scaffolds was significantly better than either uncoated scaffolds or TCP but there was no significant difference between uncoated PLLA and TCP. On the 21^st^ day, there was a big significant difference (P<0.05) between the two scaffolds, in addition, the difference in mineralization between TCP and uncoated scaffold was also significant (P<0.05). 

### Alizarin red S staining

**Figure 5:**
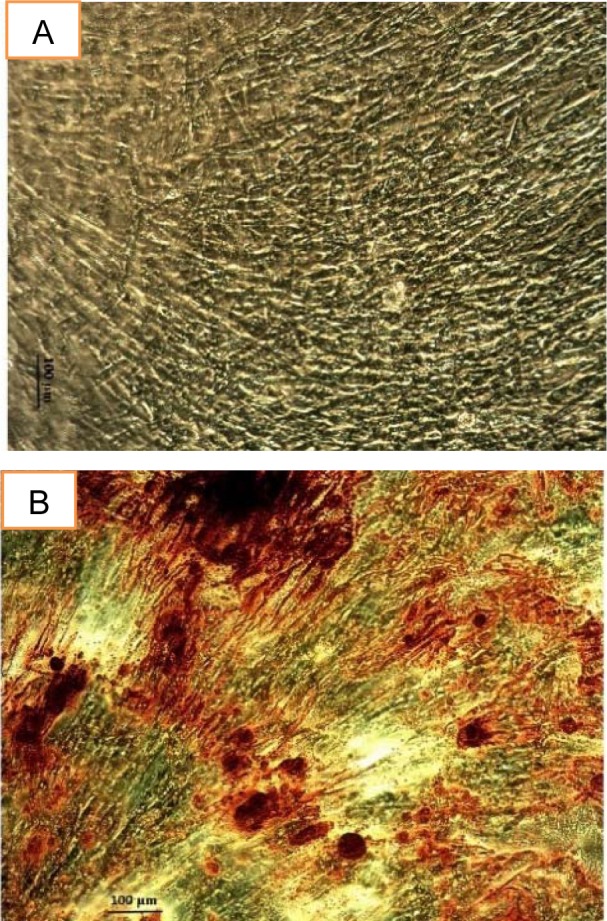
Alizarin red S staining confirming the differentiation of human MSC into the osteoblast at day 14 (A), control (B) osteogenic media

It helps to see the deposition of calcium on the extracellular matrix. The red color area after using alizarin red S staining shown in Figure 5(Fig. 5) in the osteoinduction media confirmed the osteogenic differentiation of stem cells.

See also the Supplementary data.

## Discussion

In this study, novel WJ coated PLLA electrospun nanofiber scaffold was fabricated for bone tissue engineering for the first time and the *in vitro *osteogenic potential of the scaffold was evaluated. PLLA is a biocompatible and biodegradable synthetic polymer approved by US FDA for human use. But scaffolds made from PLLA are hydrophobic and lack bioactive signals for cell recognition and attachment. Cell adhesion on the scaffold surface is crucial for cell growth, spreading, proliferation and differentiation and growth of new tissue (Balaji Raghavendran et al., 2014[7]). Different approaches were taken so far to overcome the shortcomings associated with electrospun PLLA nanofiber scaffolds. Among them the use of synthetic or natural polymers to obtain desirable mechanical properties, hydrophilicity and proper functional groups for cell attachment and recognition is the best alternative (Ardeshirylajimi et al., 2015[5]).

Wharton's jelly is a tissue containing high amounts of ECM mainly; collagen, hyaluronic acid and several GAGs (Malkowski et al., 2007[22]). Currently, surface modified electrospun nanofibers with natural polymers have attracted the attention of many researchers in the field of tissue engineering mainly because of their ECM-mimicking structure (Hoveizi et al., 2014[18]). We here hypothesized that WJ coated PLLA electrospun nanofiber scaffolds would be an ideal scaffold for bone tissue engineering under the influence of osteogenic induction. 

Collagen in WJ is the major protein component of bone ECM and is mineralized during bone formation and regeneration. It is known that collagen plays a key role in providing physical and chemical clues to retain bone function (Griffin et al., 2015[15]; Khajavi et al., 2016[19]). Coating of PLLA nanofibers with WJ have multiple benefits for tissue-engineered scaffold, because in addition to collagen, WJ is rich in a variety of fibrous proteins, interstitial proteins, and signaling molecules, including GAGs, proteoglycans, (Chan et al., 2009[9]) and peptide growth factors (Romanowicz and Bankowski, 2010[27]), all of which could facilitate cellular attachment, proliferation, infiltration, and differentiation. The surface properties of PLLA scaffold such as porosity and fiber diameter were not significantly changed by WJ coating (Seyedjafari et al., 2011[30]).

From this work, we have seen that WJ-PLLA scaffolds showed adhesion and proliferation of adipose tissue MSC much better than uncoated PLLA and TCP. This is because of the bioactivity of the components of WJ mainly of collagen which offers natural adhesion sites for cells to attach and proliferate (Chevallay and Herbage, 2000[10]).

The higher rate of cell proliferation on WJ-PLLA scaffolds than pristine PLLA scaffolds can be also the result of an increased hydrophilicity due to GAGs (hyaluronic acid) in the WJ which has an effect on cell attachment and proliferation on biomaterials. The large amount of hyaluronate makes this tissue highly hydrated (Romanowicz and Bankowski, 2010[27]).

ALP is an essential enzyme in bone matrix mineralization that breaks phosphate for calcium deposition on bone. Its activity is high before the start of calcium deposition (Anderson et al., 2004[4]). The increment in the ALP activity is taken as an early-stage marker of osteogenic differentiation of stem cells (Seyedjafari et al., 2011[30]). In this work, the activity of ALP on WJ-PLLA increased as compared with TCP and uncoated PLLA scaffolds on day 14 but decreased on the 21^st^ day, which is consistent with other works done before (Nanda et al., 2014[24]; Nguyen et al., 2012[25]). Higher ALP activity on WJ-PLLA compared to PLLA on day 14, showed the effect of WJ and its components towards osteolineage.

Calcium content or mineralization is another marker of osteogenesis but unlike the ALP activity, which is considered as an early marker of osteogenesis, mineralization is considered as a late marker of osteogenesis (Ardeshirylajimi et al., 2016[6]). As we have seen from the result, the amount of calcium deposited was significantly higher for WJ-PLLA nanofibers in comparison with TCP and PLL nanofibers in all the days tested, supporting the role of WJ in enhancement of osteogenic differentiation. 

## Conclusion

In this work, we fabricated WJ-coated electrospun PLLA nanofiber scaffolds for bone tissue engineering. Coating of WJ enhanced the osteogenic differentiation potential electrospun PLLA nanofiber scaffolds for MSCs. Coating of WJ on electrospun PLLA nanofiber scaffolds is a novel approach which totally modifies the surface properties of electrospun PLLA nanofiber scaffolds for tissue engineering application *in vitro *but further *in vivo* study is needed to confirm its use in future regenerative medicine to reconstruct large bone defects. 

## Conflict of interest

The authors declare no conflict of interest.

## Supplementary Material

Supplementary data

## References

[1] Agarwal S, Wendorff JH, Greiner A (2008). Use of electrospinning technique for biomedical applications. Polymer.

[2] Agrawal CM, Ray RB (2001). Biodegradable polymeric scaffolds for musculoskeletal tissue engineering. J Biomed Mater Res.

[3] Amjadian S, Seyedjafari E, Zeynali B, Shabani I (2016). The synergistic effect of nano-hydroxyapatite and dexamethasone in the fibrous delivery system of gelatin and poly (L-lactide) on the osteogenesis of mesenchymal stem cells. Int J Pharm.

[4] Anderson HC, Sipe JB, Hessle L, Dhanyamraju R, Atti E, Camacho NP (2004). Impaired calcification around matrix vesicles of growth plate and bone in alkaline phosphatase-deficient mice. Am J Pathol.

[5] Ardeshirylajimi A, Mossahebi-Mohammadi M, Vakilian S, Langroudi L, Seyedjafari E, Atashi A (2015). Comparison of osteogenic differentiation potential of human adult stem cells loaded on bioceramic-coated electrospun poly (L-lactide) nanofibers. Cell Prolif.

[6] Ardeshirylajimi A, Rafeie F, Zandi-Karimi A, Jaffarabadi GA, Mohammadi-Sangcheshmeh A, Samiei A (2016). Fat harvesting site is an important determinant of proliferation and pluripotency of adipose-derived stem cells. Biologicals.

[7] Balaji Raghavendran HR, Puvaneswary S, Talebian S, Murali MR, Naveen SV, Krishnamurithy G (2014). A comparative study on in vitro osteogenic priming potential of electron spun scaffold PLLA/HA/Col, PLLA/HA, and PLLA/Col for tissue engineering application. PLoS ONE.

[8] Buser D, Dula K, Belser U, Hirt H, Berthold H (1993). Localized ridge augmentation using guided bone regeneration. 1. Surgical procedure in the maxilla. Int J Periodontics Restor Dent.

[9] Chan RW, Rodriguez ML, McFetridge PS (2009). The Human umbilical vein with wharton’s jelly as an allogeneic, acellular construct for vocal fold restoration. Tissue Eng Part A.

[10] Chevallay B, Herbage D (2000). Collagen-based biomaterials as 3D scaffold for cell cultures: applications for tissue engineering and gene therapy. Med Biol Eng Comput.

[11] Daamen WF, Nillesen ST, Wismans RG, Reinhardt DP, Hafmans T, Veerkamp JH (2008). A biomaterial composed of collagen and solubilized elastin enhances angiogenesis and elastic fiber formation without calcification. Tissue Eng Part A.

[12] Duan S, Yang X, Mei F, Tang Y, Li X, Shi Y (2015). Enhanced osteogenic differentiation of mesenchymal stem cells on poly (L-lactide) nanofibrous scaffolds containing carbon nanomaterials. J Biomed Mater Res A.

[13] Glowacki J, Mizuno S (2008). Collagen scaffolds for tissue engineering. Biopolymers.

[14] Gogiel T, Bankowski E, Jaworski S (2003). Proteoglycans of Wharton’s jelly. Int J Biochem Cell Biol.

[15] Griffin KS, Davis KM, McKinley TO, Anglen JO, Chu TG, Boerckel JD (2015). Evolution of bone grafting: bone grafts and tissue engineering strategies for vascularized bone regeneration. Clin Rev Bone Miner Metab.

[16] Hao H, Chen G, Liu J, Ti D, Zhao Y, Xu S (2013). Culturing on Wharton’s jelly extract delays mesenchymal stem cell senescence through p53 and p16INK4a/pRb pathways. PLoS ONE.

[17] Hench LL, Polak JM (2002). Third-generation biomedical materials. Science.

[18] Hoveizi E, Nabiuni M, Parivar K, Rajabi-Zeleti S, Tavakol S (2014). Functionalization and surface modification of electrospunpolylactic acid scaffold for tissue engineering. Cell Biol Int.

[19] Khajavi R, Abbasipour M, Bahador A (2016). Electrospun biodegradable nanofibers scaffolds for bone tissue engineering. J Appl Polym Sci.

[20] Lin Y, Wang L, Zhang P, Wang X, Chen X, Jing X (2006). Surface modification of poly(L-lactic acid) to improve its cytocompatibility via assembly of polyelectrolytes and gelatin. Acta Biomater.

[21] Ma Z, Gao C, Gong Y, Shen J (2005). Cartilage tissue engineering PLLA scaffold with surface immobilized collagen and basic fibroblast growth factor. Biomaterials.

[22] Malkowski A, Sobolewski K, Jaworski S, Bankowski E (2007). FGF binding by extracellular matrix components of Wharton’s jelly. Acta Biochim Pol.

[23] Mattii L, Battolla B, D’Alessandro D, Trombi L, Pacini S, Cascone MG (2008). Gelatin/PLLA sponge-like scaffolds allow proliferation and osteogenic differentiation of human mesenchymal stromal cells. Macromol Biosci.

[24] Nanda HS, Nakamoto T, Chen S, Cai R, Kawazoe N, Chen G (2014). Collagen microgel-assisted dexamethasone release from PLLA-collagen hybrid scaffolds of controlled pore structure for osteogenic differentiation of mesenchymal stem cells. J Biomater Sci Polym Ed.

[25] Nguyen LT, Liao S, Chan CK, Ramakrishna S (2012). Electrospun poly (l-lactic acid) nanofibres loaded with dexamethasone to induce osteogenic differentiation of human mesenchymal stem cells. J Biomater Sci Polym Ed.

[26] Ramezanifard R, Seyedjafari E, Ardeshirylajimi A, Soleimani M (2016). Biomimetic scaffolds containing nanofibers coated with willemite nanoparticles for improvement of stem cell osteogenesis. Mater Sci Eng C Mater Biol Appl.

[27] Romanowicz L, Bankowski E (2010). Lipid compounds of human Wharton’s jelly and their alterations in preeclampsia. Int J Exp Pathol.

[28] Schofer MD, Roessler PP, Schaefer J, Theisen C, Schlimme S, Heverhagen JT (2011). Electrospun PLLA nanofiber scaffolds and their use in combination with BMP-2 for reconstruction of bone defects. PLoS ONE.

[29] Schofer MD, Veltum A, Theisen C, Chen F, Agarwal S, Fuchs-Winkelmann S (2011). Functionalization of PLLA nanofiber scaffolds using a possible cooperative effect between collagen type I and BMP-2: impact on growth and osteogenic differentiation of human mesenchymal stem cells. J Mater Sci Mater Med.

[30] Seyedjafari E, Soleimani M, Ghaemi N, Sarbolouki MN (2011). Enhanced osteogenic differentiation of cord blood-derived unrestricted somatic stem cells on electrospun nanofibers. J Mater Sci Mater Med.

[31] Shabani I, Haddadi-asl V, Soleimani M, Seyedjafari E, Hashemi SM (2014). Ion-exchange polymer nanofibers for enhanced osteogenic differentiation of stem cells and ectopic bone formation. ACS Appl Mater Interfaces.

[32] Shin H, Temenoff JS, Bowden GC, Zygourakis K, Farach-Carson MC, Yaszemski MJ (2005). Osteogenic differentiation of rat bone marrow stromal cells cultured on Arg-Gly-Asp modified hydrogels without dexamethasone and beta-glycerol phosphate. Biomaterials.

[33] Sobolewski K, Bankowski E, Chyczewski L, Jaworski S (1997). Collagen and lycosaminoglycans of Wharton’s jelly. Biol Neonate.

[34] Sobolewski K, Małkowski A, Bankowski E, Jaworski S (2005). Wharton’s jelly as a reservoir of peptide growth factors. Placenta.

[35] Woo KM, Jun J-H, Chen VJ, Seo J, Baek J-H, Ryoo H-M (2007). Nano-fibrous scaffolding promotes osteoblast differentiation and biomineralization. Biomaterials.

[36] Zhang Y, Lim CT, Ramakrishna S, Huang ZM (2005). Recent development of polymer nanofibers for biomedical and biotechnological applications. J Mater Sci Mater Med.

