# Supplementary material for: Osteogenic differentiation of mesenchymal stem cells cultured on PLLA scaffold coated with Wharton's Jelly
Source: EXCLI J. 2017 May 23;16:785–94. doi: 10.17179/excli2016-741 (PMC5547391; doi:10.17179/excli2016-741)
Supplement: Supplementary data [file EXCLI-16-785-s-001.pdf]

EXCLI Journal 2017;16:785-794 – ISSN 1611-2156
Received: October 31, 2016, accepted:May 14, 2017, published: May 23, 2017

Supplementary data to:

OSTEOGENIC DIFFERENTIATION OF MESENCHYMAL STEM CELLS CULTURED ON PLLA SCAFFOLD COATED WITH WHARTON'S JELLY

Marziehsadat Ahmadi[a], Ehsan Seyedjafari[b*], Seyed Jalal Zargar[a], Gebremariam Birhanu[c,d], Ali Zandi-Karimi[b], Bahareh Beiki[b], Kadriye Tuzlakoglu[e]

[a] Department of Cell and Molecular Biology, School of Biology, College of Science, University of Tehran, Iran
[b] Department of Biotechnology, College of Science, University of Tehran, Iran
[c] Department of Pharmaceutics, Faculty of Pharmacy, Tehran University of Medical Sciences, International Campus (TUMS-IC), Tehran, Iran
[d] School of Pharmacy, College of Health Sciences, Addis Ababa University, Ethiopia
[e] Department of Polymer Engineering, Yalova University, Turkey

* Corresponding author: Ehsan Seyedjafari, Ph.D., Department of Biotechnology, College of Science, University of Tehran, Iran; E-mail: seyedjafari@ut.ac.ir; Tel, Fax: +98-21-66491622

http://dx.doi.org/10.17179/excli2016-741

This is an Open Access article distributed under the terms of the Creative Commons Attribution License (http://creativecommons.org/licenses/by/4.0/).

Supplementary Table 1: The raw data obtained from MTT (Figure 2), ALP activity (Figure 3) and total calcium content (Figure 4) assays

	TCPS			PLLA			PLLA/WJ		
**MTT (OD)**									
**Day 1**	0.392	0.388	0.31	0.367	0.326	0.275	0.452	0.484	0.422
**Day 4**	0.511	0.461	0.502	0.501	0.461	0.438	0.579	0.587	0.641
**Day 7**	0.639	0.58	0.505	0.543	0.53	0.604	0.608	0.687	0.692
**Total Calcium Content (µ/well)**									
**Day 7**	14.97914	15.04536	14.8467	14.64804	13.85339	15.01225	17.25038	19.3166	18.13767
**Day 14**	21.0651	24.97212	24.04503	20.63466	22.95239	22.62128	26.21727	27.3166	25.65439
**Day 21**	34.11695	37.36176	35.97113	28.25641	31.17012	33.52096	36.93133	35.60691	40.50725
**ALP Activity (IU/mg total protein)**									
**Day 7**	0.595	0.565	0.523	0.474	0.429	0.463	0.437	0.526	0.4605
**Day 14**	0.8645	0.648	0.997	0.843	0.676	0.7155	1.012	0.879	0.9555
**Day 21**	0.475	0.775	0.5425	0.473	0.633	0.461	0.644	0.578	0.527

TCPS: Tissue culture polystyrene, PLLA: Poly-L-lactic acid, WJ: Wharton's jelly, ALP: Alkaline phosphatase
